# Australian perspectives towards genomics informed risk-based cancer screening programs: a focus group study

**DOI:** 10.1007/s12687-026-00971-y

**Published:** 2026-11-07

**Authors:** Deborah Di-Xin Zhou, Amber Salisbury, Renee Mostyn, Kate L. A. Dunlop, Karen Canfell, Louisa Collins, Michael Caruana, Cynthia Low, Anne E. Cust, Richard Norman, Julia Steinberg, Amelia K. Smit, Alison Pearce

**Affiliations:** 1 NHMRC Clinical Trials Centre, University of Sydney, Sydney, Australia; 2 The Daffodil Centre, The University of Sydney, and Cancer Council NSW, Sydney, Australia; 3 Sydney School of Public Health, The University of Sydney, Sydney, Australia; 4 QIMR Berghofer Medical Research Institute, Brisbane, Australia; 5 Melanoma Institute Australia, The University of Sydney, Sydney, Australia; 6 Curtin University, Perth, Australia; 7 Viertel Cancer Research Centre, Cancer Council Queensland, Brisbane, Australia; 8 Lived Experience Expert / Health Consumer, Sydney, Australia

**Keywords:** Genomic testing, Cancer, Risk-tailored, Risk-stratified, Screening, Focus groups, Polygenic risk scores, PRS, Qualitative, Focus groups

## Abstract

**Background:**

Predictions of cancer risk based on genomic data (polygenic risk scores) can stratify a population into different risk groups, allowing risk-tailored cancer screening programs. While such approaches are generally viewed as acceptable, there remain gaps in understanding Australian perceptions to guide implementation of future cancer control programs and policies. This project aimed to explore Australians’ views on using genomic information to tailor cancer screening and how such programs could be implemented.

**Methods:**

Four focus groups (*n* = 28) were held using a discussion guide that explored key features at each stage of potential programs: deciding to take the test, completing the test, receiving the results, and the subsequent impact on screening. Reflexive thematic analysis was used to identify, code and report themes.

**Results:**

Three central themes emerged: (1) The value of knowing, which reflected motivations to test for personal as well as family and social benefits; (2) Navigating the complexity of genomics, highlighting challenges in understanding potential pre-test information and results; and (3) Trust in testing, providers, and government which influenced program acceptability for the initial genomic testing. However, this trust may not always result in participants following subsequent cancer screening recommendations.

**Conclusion:**

Our study identifies the need for community education and clear communication about genomics-informed risk-tailored cancer screening. Potential screening programs should consider the role of healthcare professionals and ensure they receive appropriate training to meet public needs. Further research should explore potential adherence to risk-tailored screening recommendations.

**Supplementary Information:**

The online version contains supplementary material available at https://doi.org/10.1007/s12687-026-00971-y.

## Introduction

Cancer screening enables early detection and prevention, improves prognosis and reduces health care costs and long-term impacts of treating later-stage disease (Bretthauer and Kalager 2013). In Australia, population-based national screening programs are guided by the *Population-based screening framework *(2018). Currently, established programs exist for breast, cervical, colorectal, and lung cancer (for current or past smokers without symptoms), while opportunistic early detection is used for prostate cancer and melanoma. With the exception of lung cancer screening, the population-based screening programs offer a ‘one size fits all’ approach, with age being the main criterion for determining screening eligibility (Department of Health 2023). In risk tailored screening, different aspects of the screening program can be modified on the basis of an individual’s cancer risk.

An individual’s cancer risk may be estimated using demographic, behaviour, and/or genomic information including polygenic risk scores (PRS). PRS capture a person’s risk for a condition by combining information from many genetic risk variants (Young et al. 2023). In this paper, we classify PRS as a form of genomic testing. Unlike genetic testing for rare, highly penetrant genetic variants that strongly predispose to disease (e.g., pathogenic *BRCA1/2* variants), PRS testing considers a large number of genetic variants with individually modest effects. However, when aggregated, these variants can collectively explain a significant proportion of disease susceptibility. These scores are available for common cancers such as breast, colorectal, melanoma and prostate (Schmit et al. 2019; Landi et al. 2020; Zhang et al. 2020; Conti et al. 2021).

There are several ways screening programs could be tailored to an individual’s PRS, including adjustments to start age, screening technology, and screening frequency (Dent et al. 2013). For example, a woman at high risk of breast cancer might start screening at a younger age, screen more often, and/or use breast magnetic resonance imaging rather than standard mammography. Modelling studies have demonstrated the potential of such programs to enhance cancer detection and reduce mortality (Jia et al. 2020; Huntley et al. 2023). At the moment, population-based use of PRS is still largely confined to research settings, including randomised controlled trials that evaluate the clinical impacts of integrating PRS into cancer screening programs (Brooks et al. 2021; Roux et al. 2022), such as the Australian SCRIPT trial for colorectal cancer (Saya et al. 2022a, b) and the United States WISDOM trial for breast cancer (Esserman et al. 2025). These trials are expected to help establish the potential of PRS in supporting risk-tailored screening programs, alongside assessments of broader factors such as disease burden, cost-effectiveness, and public acceptability. Integration of PRS into clinical cancer risk assessment is also being investigated in the US Electronic Medical Records and Genomics (eMERGE) Network study (Lawson et al. 2026; Limdi et al. 2026). Looking into the future, population-based use of PRS is explicitly included in NHS England’s 10-year plan (2025) as part of a new “genomics population health service”, and the Australian National Framework for Genomics in Cancer Control (Cancer Australia 2025) action aims to “embed evidence-based culturally safe genomic testing into national cancer screening programs, to enable risk-stratified screening”, stating that “In the near future, it is likely PRS may be used as a tool in conjunction with traditional risk factors for population-based risk stratification and wider genomics-informed cancer care”.

Globally, there is evidence to support the public acceptability of genomics-informed risk-tailored cancer screening, with studies highlighting several potential benefits. Many individuals are motivated to better understand their cancer risk to support proactive management, whilst also seeing the logic and wider societal benefits of such programs (Smit et al. 2020; Smith-Uffen et al. 2021; Taylor et al. 2023). However, concerns have been raised about clinical utility, cost, privacy, confidentiality, psychological impact, and effects on families.(Smith-Uffen et al. 2021) Previous Australian qualitative studies have examined risk-tailored screening generally (Dunlop et al. 2023) and single-cancer genomic programs, (Lippey et al. 2019; Smit et al. 2020; Saya et al. 2022a, b) with similar findings. There is general acceptance of moving to more individualised risk stratification, including the use of genomic information, but adequate communication and workforce planning would be key to successful implementation (Lippey et al. 2019; Smit et al. 2020; Saya et al. 2022a, b; Dunlop et al. 2023). However, there has been no qualitative exploration of public perspectives for the wider use of genomic risk assessment, possibly including multiple cancers. This gap is particularly relevant given recent quantitative evidence suggesting public preference for more comprehensive testing approaches that assess risk for multiple cancers rather than a single cancer (Venning et al. 2022a, 2022b, Saya et al. 2022a, b). This study is the first qualitative study to explore Australian public understanding and opinions regarding genomic testing for risk-tailored screening programs that could potentially assess risk across multiple cancers.

## Methods

### Participant recruitment

We aimed to conduct three to four focus groups of six to eight adults to allow for comprehensive exploration of all relevant issues based on previous research (Hennink et al. 2019; Hennink and Kaiser 2022). Eligible participants were adults aged 30 to 80 years old, living in Australia, without an active cancer diagnosis (i.e. not diagnosed with cancer within the past year) and able to speak English sufficiently to participate in a focus group discussion. Participants for the focus groups (offered face to face or for those outside Sydney online via Zoom) were recruited through a panel provider (Taverner Researcher Group) using maximum variation sampling, which is a technique that aims to collect data from a wide range of perspectives (Palinkas et al. 2015). The panel provider sent invitation packs to potentially eligible participants in a range of urban, outer urban and regional areas in New South Wales, Victoria and Queensland to capture diversity in levels of sociodemographic, educational and health literacy characteristics. The invitation pack contained a Letter of Introduction, Participant Information Sheet, Consent Form, and details of the focus group locations, dates, and times. Potential participants responded via email. The following sociodemographic data was collected by the panel provider to capture areas of diversity in recruitment: living location (metropolitan/regional), gender, age, Aboriginal or Torres Strait Islander status, level of education, employment status, history of cancer, participation in previous cancer screening or genomic/genetic testing, and languages spoken. All participants provided written informed consent. Participants were provided with a $75 gift voucher to compensate them for their travel time and expenses after the focus groups were conducted.

Ethics approval was obtained from The University of Sydney Human Research Ethics Committee (2022/844). The reporting of this focus group study follows the consolidated criteria for reporting qualitative research checklist (COREQ) (Tong et al. 2007).

### Data collection

Focus groups were conducted by an experienced facilitator trained in conducting focus groups (AS) with an experienced observer (AP) to aid the facilitator and record relevant observations. The one-hour focus groups were informed by a discussion guide to identify the values, beliefs, and opinions of the Australian community with regards to genomic testing to support risk-tailored screening programs. The discussion guide (Supplementary 1) included an introduction to genomics and risk-based cancer screening, followed by a discussion of genomic testing implementation options (e.g. testing location, health professionals involved in risk communication). These topics were selected based on a systematic review of discrete choice experiments (DCE) in genomics-informed chronic disease screening, (Salisbury et al. 2024) two unpublished background reviews conducted by the research team in similar areas, and consultation with stakeholders (Supplementary Fig. 1). Participants were also given opportunities to discuss concepts which were not listed. The discussion guide was refined iteratively after each focus group to maximise clarity.

### Data analysis

The focus groups were audio recorded and transcribed verbatim by AS. Following Braun and Clarke’s methods for reflexive thematic analysis, (Braun and Clarke 2006, 2019, 2021a, b; Braun et al. 2014) the team initially spent time familiarising themselves with the dataset, including recordings, transcripts, observer notes, and outputs of focus group activities. The transcripts were then double coded independently (by DZ, RM and AS) using NVivo (Lumivero 2023) and taking an iterative approach that included regular discussion (with AP and AKS), and reflection by the multidisciplinary research team (including expertise in public health, economics, clinical trials, and genomics). This process was used for the initial coding, to generate themes and to finalise the central concept for each theme (Braun and Clarke 2019, 2021a, b). The 15-point Thematic Analysis checklist guided the identification of patterns and development of themes within the data (Braun and Clarke 2024).

We adopted an experiential approach underpinned by a reflective view of language and focussed on the participants perceptions, perspectives, and experiences (Braun et al. 2014). Although no predefined coding framework was used, the approach was predominantly deductive as we used our existing research (Salisbury et al. 2024) as a lens through which we analysed and interpreted our data.

## Results

### Participant demographics

We conducted four focus groups (two in-person and two via Zoom) with a total of 28 participants (Table 1). Over half of participants were male, half were aged 50–69 years old, and three quarters lived in a metropolitan area. One-third held an undergraduate degree, while only 14% held a postgraduate degree. Similar numbers reported a history of cancer more than a year ago (14%) or previously undergoing genomic or genetic testing (14%), while 79% reported having participated in any cancer screening. After four focus groups we were confident that we had comprehensive exploration of the relevant issues.

**Table 1:** Baseline demographics

Focus group	Group 1	Group 2	Group 3	Group 4	Total
Demographic	*N* = 7	*N* = 6	*N* = 7	*N* = 8	*N* = 28
Region (mode)	Outer Sydney (In person)	Inner Sydney (In person)	Regional (Online)	Metro Melbourne/Brisbane (Online)	
Gender					
Female	3	2	3	4	12
Male	4	4	4	4	16
Age					
< 50	2	3	2	3	10
50–69	4	2	5	3	14
≥ 70	1	1	0	2	4
Aboriginal or Torres Strait Islander	0	0	0	0	0
Education					
School or High school certificate	1	0	3	2	6
Trade	2	3	1	2	8
Undergraduate	3	3	1	3	10
Postgraduate	1	0	2	1	4
Employment					
Full time	2	5	5	3	15
Part time	2	0	0	1	3
Self employed	1	0	0	1	2
Retired	1	1	1	3	6
Casual	0	0	1	0	1
Unemployed	1	0	0	0	1
History of cancer					
Yes	1	1	1	1	4
No	6	5	6	7	24
Cancer screening participation					
Yes	5	5	6	6	22
No	2	1	1	2	6
Previous genomic/genetic testing					
Yes	1	1	1	1	4
No	6	5	6	7	24
Languages					
English	7	4	3	6	20
Other in addition to English	0	2	4	2	8

### Themes

**Fig. 1:**
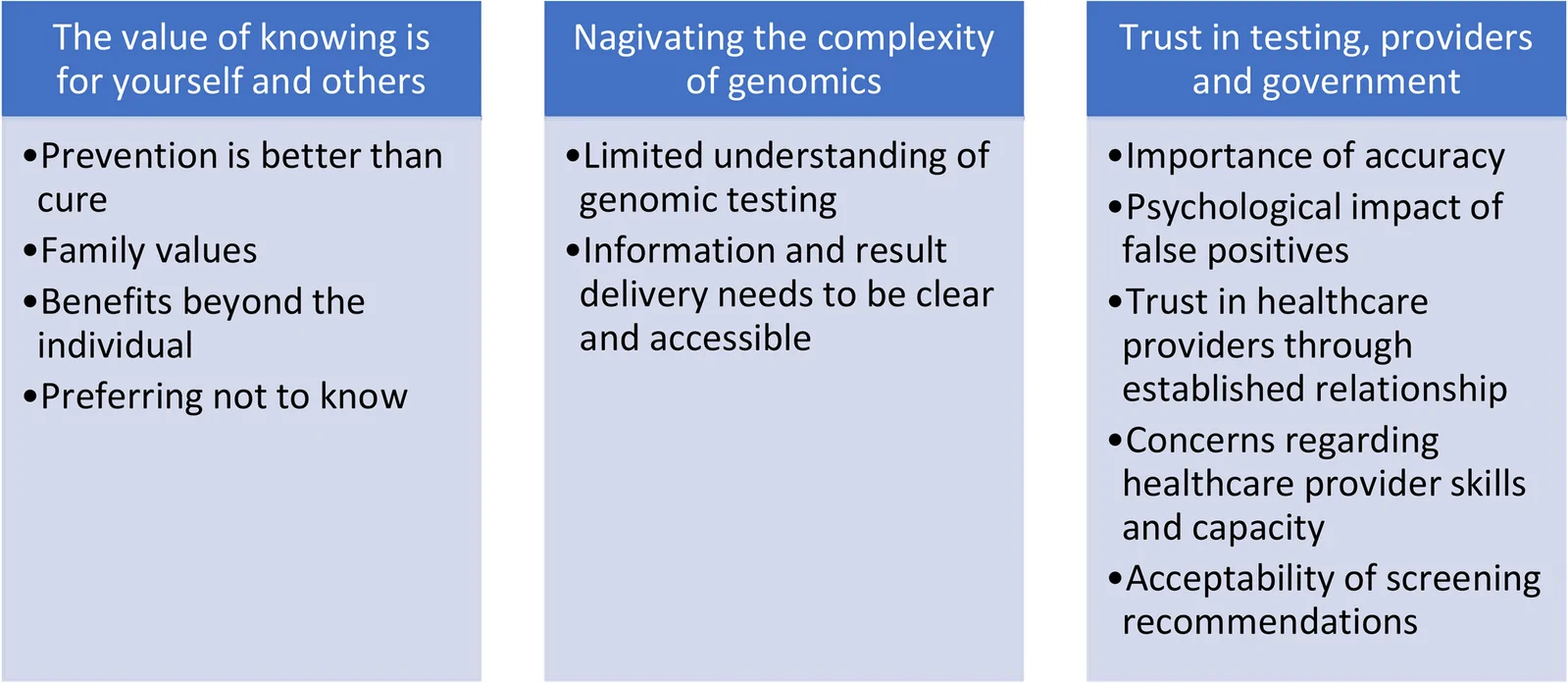
Themes and sub-themes

Three themes were generated: (1) the value of knowing is for yourself and others, (2) navigating the complexities of genomics, and (3) trust in testing, providers and government (Fig. 1). The themes, sub-themes and illustrative quotes are presented below, and additional quotes can be found within the Supplementary materials (Table S1).

#### The value of knowing is for yourself and others

The majority of participants demonstrated strong interest in genomic testing for cancer risk, with some enquiring about immediate testing opportunities during the focus group sessions. While individuals recognised the personal benefits, they also highlighted benefits to their family and the broader community.

##### Prevention is better than cure

Participants valued the potential to take charge of their health by participating in a genomics informed cancer screening program and reduce their cancer risk through increased screening if found to be at high risk. Many stated they would also modify behaviour, such as diet and exercise, assuming this would reduce overall risk based on the genomic test results.

*Female focus group 4 (Pt ID 21)*: I’d be very keen to do something like this… I’d be all for having this testing done independently if the GP wasn’t even that fussed about it. I’m quite excited about it.

##### Family values

The value of knowing was also driven by a participant’s personal need to live longer for family or to positively influence their family’s attitudes and behaviour towards health and prevention.

*Female, focus group 1 (Pt ID 3)*: My first decision would be myself and then you know, the children and society. All that because like you're a father [speaking to another male participant], you've got a family.

##### Benefits beyond the individual

Some participants also expressed an altruistic approach to genomic testing, believing they are contributing to the greater good.

*Male, focus group 1 (Pt ID 6)*: Also something like this [genomics-informed risk-based cancer screening] will basically benefit the whole society, right? Not just the few individuals, right. So it definitely will be a big impact because in long term it could affect plenty of people, to save lives basically.

##### Preferring not to know

Some participants suggested other members of the public might not undertake genomic testing if they do not believe in the benefits of cancer screening, are sceptical of traditional ‘western’ concepts of medicine, hold views of genetic determinism, or intentionally choose not to know about their risk. However, no participants expressed these views for themselves during the focus groups.

*Male, focus group 2 (Pt ID 12)*: I’ve got a few friends that are sceptics and anti vaxxers and that’s their choice but like there is a smaller minority that are probably a bit sceptical about results and these sorts of tests and aren’t interested.

#### Navigating the complexity of genomics

##### Limited understanding of genomic testing

During discussions, most participants confused PRS-based genomic risk (where multiple genetic variants each contribute small effects to overall risk) with monogenic conditions (where pathogenic variants in one gene strongly influence disease risk) at times. Several participants mistakenly described genomic testing as a diagnostic test and equated receiving results with a cancer diagnosis.

*Female, focus group 4 (Pt ID 22)*: Most people who get a cancer diagnosis, they sort of go into shock and they're not really taking in the information that the doctor's giving them.

There was also limited understanding of overdiagnosis and overtreatment, as well as misconceptions regarding test scope and accuracy. Some participants incorrectly assumed that genomic risk testing would not provide information beyond what they could learn from family history.

*Male, focus group 4 (Pt ID 25)*: I think we already know our genetics from our family histories and things like that.

##### Information and result delivery needs to be clear and accessible

Participants emphasised the importance of delivering information and results in a way that is clear, accessible, and culturally appropriate. Digital tools, such as telehealth or online results, were viewed positively to increase accessibility.

*Male, focus group 3 (Pt ID 14)*: I like it online only because I have a brain injury and I sometimes I have to read things 3, 4, 5 times to get the information through to my head and understand it.

*Male, focus group 1 (Pt ID 5)*: Yeah, I think it should be simple to understand. Like if it's too technical, too medical, it’s hard to understand for everyone.

They valued the option of consulting with a health professional (telehealth or in person), particularly when receiving high-risk results.

*Female, focus group 4 (Pt ID 21)*: If something comes back [high risk result], I think you need to be guided by the health professional, so you don't get paranoid and think you're going to die and all this sort of stuff.

#### Trust in testing, providers and government

Discussions primarily focused on the delivery of the genomic test results, assuming healthcare providers would be responsible for this. There was less discussion about other program components (such as invitations, reminders, and source of screening recommendations), and government agencies responsible for these components were not explicitly mentioned.

##### The importance of accuracy

Participants raised accuracy as an important factor in deciding whether they would use genomic information to guide cancer screening. There was also an expectation that any genomic testing implemented in clinical practice would meet high standards of accuracy, likely reflecting an underlying trust in government-led regulatory oversight.

*Male, focus group 3 (Pt ID 14)*: I would have thought that the tests wouldn't have been made available unless they were extremely accurate... Sort of like a like a 95% sort of thing or the in that area.

While many preferred genomic testing for multiple cancers and diseases, some participants worried this comprehensive approach might compromise accuracy or affordability.

*Male, focus group 1 (Pt ID 6)*: It would be nice to get [a single test for multiple cancers], but we need to find some reasonable compromise, what you could realistically do here without losing accuracy?

##### Psychological impact of false positives

Accuracy was particularly valued by those who had previous personal history of false positive test results, who described detrimental psychological consequences. Other clinical consequences such as overtreatment and overdiagnosis were not mentioned.

*Male, focus group 4 (Pt ID 23)*: Accuracy is very important. I was screened for Alzheimer's, I was told I had it. It caused enormous distress in the family and I ultimately, I don't have it. I can't tell you the drama that it was involved in it.

##### Trust in healthcare providers through established relationships

Participants who had longstanding relationships with their GPs tended to trust their skills and knowledge and were willing to engage with them as part of the genomic testing and screening program.

*Female, focus group 4 (Pt ID 26)*: I like to go to my GP because he knows us.

##### Concerns regarding healthcare provider skills and capacity

However, other participants raised concerns about the competence of both GPs and nurses in genomics, their priorities, workload capacity, and interest in genomic testing. Further education and upskilling were identified as key requirements for any health care providers involved in a potential testing and screening program. There was a general lack of awareness about genetic counsellors.

*Female, focus group 2 (Pt ID 8)*: Are the GP's going to be educated about what this is [genomics informed risk cancer screening], why we’re doing it and what their role is?

*Female, focus group 2 (Pt ID 13)*: I cannot see practise nurse sitting down going ‘ohh, let's take a look at your genomic test’. They're not trained to do that.

*Male, focus group 4 (Pt ID 23)*: A new occupation has been created [genetic counsellors] (laughs).

##### Acceptability of screening recommendations

The majority of participants indicated they would follow recommendations for increased screening if at high cancer risk.

*Female, focus group 2 (Pt ID 8)*: I would be [in response to being asked whether they would follow the recommendations for increased screening]. I’m a rule follower. Yes, so that’s just the way that I’m geared.

Many participants described that they felt they might not comply with recommendations to reduce screening frequency if found to be low risk.

*Female, focus group 3 (Pt ID 15)*: Even if I was told that I was low risk, I would still do the screening.

*Male, focus group 3 (Pt ID 14)*: I’m exactly the same… I’d rather keep on top of the small percentage and have tests as regularly as I can, but it’s good to know that you’re at low risk.

## Discussion

The Australian population-based screening framework requires that any implemented screening program is acceptable to the target population (Department of Health and Ageing 2018). This study provides evidence of how the Australian community perceives potential genomics-informed risk-based cancer screening. The majority of participants displayed a straightforward acceptance of genomic testing within this context. Three overarching themes emerged from the focus group discussions: (1) The value of knowing, which reflected motivations to test for personal as well as family and social benefits; (2) Navigating the complexity of genomics, highlighting challenges in understanding potential pre-test information and results; and (3) Trust in testing, providers, and government which influenced program acceptability. These three themes can inform potential components of a genomics-informed risk-based cancer screening program in Australia, from invitations, to doing the genomic test, to return of screening recommendations, and these implications are discussed below.

Despite participants being interested in undergoing genomic testing, this study found that genomic information and risk were complex concepts to communicate and understand. Some participants did not understand the differences between screening and diagnostic tests and between PRS and single highly penetrant genes. This led participants to incorrectly estimate their personal genomic cancer risk from their family history of cancer - findings that align with the broader literature in cancer screening and genomic testing (​Dent et al. 2013; Lautenbach et al. 2013). Furthermore, there was a lack of awareness and understanding of overdiagnosis and overtreatment. These represent major personal and societal harms of cancer screening programs, and are well-documented outcomes in cancer screening (Croswell et al. 2010). The potential limitations of PRS use, such as reduced predictive performance for some ancestry groups (Martin et al. 2019), uncertainty of real-world costs and effects of PRS-informed risk-based screening (Dixon et al. 2022), and short-term distress at risk communication (Wallingford et al. 2023; Dannhauser et al. 2024) were rarely raised. The misunderstandings exhibited by participants highlight the need for easily understandable information that provides education on risk-based screening (including potential benefits and harms of both PRS-informed risk assessment and of risk-based screening) to support informed decision-making in potential future genomic risk assessments.

In response to the complexity of genomics, participants expressed mixed views about how the results should be delivered. However, there was consensus on the need to leverage health technology whilst offering the option to consult with a healthcare provider. A recent quantitative survey investigated three different approaches to the PRS cancer screening process in Australia: (1) an online process, where the test is ordered and taken at home, with results provided online; (2) visiting a GP for both the test and results; and (3) obtaining a referral to a genetic specialist, who administers the test and delivers the results (Venning et al. 2022a, 2022b, Saya et al. 2022a, b). They found a positive preference for the GP process and negative preference for the specialist process, compared to the online process. Our study builds on these findings by suggesting preferences may vary depending on the program component. For instance, the requirement to obtain a referral and see a specialist for the initial decision to undergo genomic testing could explain the overall negative preference for that process, while specialists may still be favoured for delivering results. Our focus group participants expressed concerns about GPs and practice nurses having limited genomics knowledge, concerns that GPs themselves share (Smit et al. 2019). Given the shortage of genetic counsellors and their role in managing rare but highly penetrant genes rather than in screening, primary care providers will likely play a role in genomics-informed risk-based cancer screening. These findings highlight the need to upskill primary care providers and provide flexible approaches to delivering genomic test results that can accommodate diverse patient needs.

Our study found that the acceptability of genomics-informed risk-based cancer screening program will depend on trust in healthcare providers and government agencies. To ensure feasibility and cost-effectiveness, such programs will likely balance intensive screening for high-risk individuals with reduced screening for those at low risk (Wong et al. 2021). In a nationally funded program, this would mean reducing access to reimbursed tests for low-risk individuals, who may be able to request to pay out-of-pocket if they wish to undergo additional testing outside the screening program. The concerns raised by participants about reducing screening align with qualitative interviews in Australia regarding individualised breast cancer screening models, where women expressed strong emotional ties to the current structure (Lippey et al. 2019). Similar findings have been observed in breast cancer screening internationally, (Ghanouni et al. 2020) and in the broader context of risk-tailored screening across various cancers, including where genomics was not considered (Dunlop et al. 2024). Within our study, we found trust was a key factor in whether or not participants expressed acceptance towards the recommendations. Trust was often based on confidence in the expertise or long-term relationships with healthcare providers who were assumed to deliver the results, despite it being government agencies who make the recommendations. Similarities can be drawn to the recent changes to cervical cancer screening, where many countries, including Australia, updated guidelines to extend the interval between pap smears and replace them with human papillomavirus testing (Department of Health 2025). While some stakeholders initially raised concerns about reduced screening frequency, women later reported trusting that government agencies have made changes for the better - a trust reinforced through conversations with their healthcare providers (Dodd et al. 2020). Together, this suggests the acceptability of genomics-informed risk-based cancer screening recommendations will likely rely on strong trust in healthcare providers and government-led public health campaigns that clearly communicate the evidence underpinning such approaches.

Our study has several strengths. The use of focus groups and thematic analysis enables a rich and nuanced exploration of Australian participants’ perspectives. There was generally good engagement, with participants open to sharing personal experiences and generally receptive to alternative views. There are also some potential limitations. Although our study found high interest in genomic testing, actual uptake is influenced by multiple factors that were not discussed, as described by the Information-Motivation-Behavioural Skills model (Fisher et al. 2003). Additionally, selection bias is likely present due to the method of recruitment, which means individuals may be more likely to self-select to participate in the study if they have an interest in the topic, and may also be ‘professional’ participants who are experienced with focus group discussions. This may mean the views expressed are from people who are more engaged in the topic than the general population. Self-reported cancer screening behaviours were collected, but these may be subject to recall bias, limiting our ability to explore how previous screening behaviour influenced findings. One zoom focus group was less engaged, with limited interaction evidenced with the use of voice (rather than video) and chat function (rather than speaking) and may mean results are biased towards the face-to-face focus groups, who tended to be more urban-based. The focus group methodology is also limited by more assertive participants who may dominate conversation whilst those less assertive will struggle to give their opinion (Smithson 2000), although this was mitigated with the use of a skilled facilitator. The study had no participants who identified as Aboriginal or Torres Strait Islander and only recruited English-speaking participants, which limits the generalisability of the results.

The insights from this work can offer broader guidance to international strategic frameworks such as the UK’s Genomic Medicine Service (NHS 2025), Europe’s Beating Cancer Plan (European Commission 2022), and the US Cancer Moonshot (2016. Our study highlights the importance of community education to address the complexities of genomics-informed risk-based screening programs, including its potential benefits and harms. Clear communication is needed to distinguish between risk assessment tests, cancer screening, and diagnostic tests. When designing potential programs, features such as the involvement of trusted healthcare providers and provision of training should be carefully considered to ensure public acceptability. Perspectives on process and logistical features such as the amount of information received and healthcare provider interactions varied across all components of the potential program, from initial decision-making to receiving results to the subsequent cancer screening recommendations. Further in-depth research is needed to identify Australian preferences for specific aspects of potential genomic risk assessment and risk-based screening programs, to examine how different features would impact the uptake and equitability of hypothetical programs, and to explore whether screening recommendations would be accepted.

## Supplementary Information

Below is the link to the electronic supplementary material.

Supplementary Material 1 (DOCX 96.0 KB)

## Data Availability

No datasets were generated or analysed during the current study.
