# Supplementary material for: Australian perspectives towards genomics informed risk-based cancer screening programs: a focus group study
Source: J Community Genet. 2026 Nov 7;17(6):137. doi: 10.1007/s12687-026-00971-y (PMC13744575; doi:10.1007/s12687-026-00971-y)
Supplement: Supplementary file 1 [file 12687_2026_971_MOESM1_ESM.docx]

**Supplementary Information 1: Focus group discussion guide**

*Welcome*

Good morning/afternoon everyone,

My name is xx, I’m a researcher at the Daffodil Centre, and I will be facilitating this focus group. Just a reminder that taking part is voluntary. You are free to stop at any stage or to not answer any of the questions.

Before we begin, I would like to acknowledge and pay respect to the traditional owners of the land on which we meet, the Gadigal people the Eora nation. It is upon their ancestral lands that the University of Sydney is built.

*Overview of topic*

I will now briefly describe the topics we will cover today. We will be discussing cancer screening, and what do I mean by that? Well cancer screening involves tests that look for early signs of cancer or the conditions that cause cancer. Some examples of cancer screening tests include mammograms looking for breast cancer, skin checks looking for melanoma, blood tests looking for prostate cancer, or tests of poo looking for bowel cancer.

We’ll now look at breast cancer screening in a bit more detail. Current breast cancer recommendations are based on age, and target women between 50 and 74 years old. Women who are eligible will have a screening mammogram every two years. If symptoms are detected during screening, then they will go on to have a diagnostic mammogram as a separate step.

Current cancer screening programs do not take personal genomic risk into account. We are interested in whether cancer screening recommendations could be tailored based on an individual’s genomic risk.

Now what do I mean by genomics? So genes can influence someone’s chance of developing cancer. You might have already heard of genetic testing, which tests a small number of specific genes, such as BRCA testing for breast cancer. This is different to genomic testing. A genomic test can test a large number of genes each with a small impact on your risk, and when taken together these genes can have a very large impact.

Now to look at how genomic testing could happen in practice. First, you would make a decision to have a test and learn more about their risk. You would then have a genomic test, which involves providing a small saliva sample in a collection tube or giving a blood sample. The sample then gets tested at the lab. Depending on how the testing would work, the results could be sent directly to you, or to your healthcare provider. Based on these results, your cancer screening recommendations might be adjusted. For example, people at higher risk of developing cancer may start screening at a younger age, screen more frequently, or have a different type of screening test. In contrast, people at a lower risk of developing cancer may screen less frequently.

Currently we don’t know what the public thinks about using genomics in screening programs. That’s why we want to understand what you think about using genomic information to tailor cancer screening. We will be focusing on colorectal cancer, breast cancer, prostate cancer and melanoma.

*Housekeeping*

Before we begin the discussion, I’ll just mention some housekeeping points. There are no wrong answers but rather differing points of view. Please feel free to share your point of view even if it differs from what others have said. Keep in mind that we're just as interested in negative comments as positive comments. We are also interested in hearing everyone’s opinions, so please don’t speak over each other. Today my role is to moderate the discussion and to listen what you have to say. I encourage you to look at each other when speaking and respond to each other’s comments rather than directing responses to me.

We are recording the session because we don't want to miss any of your comments. People often say very helpful things, and we can't write fast enough. We will be on a first name basis today, but we won't use any names in our reports.

Let’s get started with a warm-up activity and then I’ll give you some questions to start the discussion, and make sure we get views from everyone within the hour.

| **Section** | **Description** | **Key questions** | **Prompts** |
| --- | --- | --- | --- |
| Warm up1 – ice breaker | Warming up the participants by getting them to introduce themselves and establishing a relaxed environment | Can everyone please introduce themselves | What is your name and what do you spend most your time doing? |
| Warm up 2 – introduction to topic | Warming up the participants by introducing them to the topic | Raise your hand if you have ever participated in cancer screening. | What type of test/screening was it?  Would any of you be happy to share what the test was for? No worries if you would rather not. |
| Section 1 | Discuss each feature on the list and to the wording of features.  Work through the steps one by one | What does [features] mean to you?  Which of these are important to you and why?  Which features do not seem important to you and why?  Do you think any of the features are less or more important when looking at breast cancer, prostate cancer, colorectal cancer or melanoma?  Are there any features not listed that you would consider important? | What wording would make the features easier to understand?  How would feature X influence your decision to take part in the genomic testing/screening program? |
| Section 2 | Each person will be asked to choose the 3 most important features and the least important feature. | What three features are most important to you and which feature is least important? | Which feature ranking do you not agree with? |
| Final question | Last question to gather any extra information | Did anyone’s views change through the discussion, if so how?  Is there anything else you would like to add to this discussion? | What changed your mind? |

I would now like you all to have a look at the handout I have provided you. This is a diagram showing the stages of testing. In the arrow you see the stages as deciding to have the test, doing the test, receiving the results and how the results impact on screening.

Below the arrow, there are features of the screening which you may think about at each stage. The box at the bottom has process features listed which may be relevant to every stage.

Today we will be discussing each feature listed, working through the stages one by one. We will first focus on the ‘Decide’ stage.

Take some time to read the features specific to the decide stage and the process features listed in the box at the bottom while thinking about the decision to undergo testing. When you are ready, we will discuss what the features mean to you and whether or not you think they are important.

*What does [features] mean to you?*

*Which of these are important to you and why?*

*Which features do not seem important to you and why?*

*Do you think any of the features are less or more important when looking at breast cancer, prostate cancer, colorectal cancer or melanoma?*

*Are there any features not listed that you would consider important?*

Extras:

*What wording would make the features easier to understand?*

*How would feature X influence your decision to take part in the genomic testing/screening program?*

**Specifics**

*What does accuracy mean to you?*

*Which feature do you think is most important out of the process features?*

*How do you feel about those with a lower risk result being given a lower frequency of screening?*

*Would you be likely to follow the screening recommendations?*

*Would the result of the screening test impact on how you would want to receive the results? If so, how?*

**Supplementary Figure 1: Attributes related to genomic testing used in focus group discussions**

**Supplementary Table 1: Themes, sub-themes and quotes**

| THEME | QUOTES |
| --- | --- |
| Theme: The value of knowing is for yourself and others | |
| Prevention is better than cure | *Female, focus group 4 (Pt ID 21):* I'd be very keen to do something like this, especially seeing as I've just had a Melanoma removed and I've had some issues with colonoscopies. I'd be all for having this testing done independently if the GP wasn't even that fussed about it. I'm quite excited about it.  *Female, focus group 4 (Pt ID 13)*: You know prevention is better than cure. |
| Family values | *Female, focus group 1 (Pt ID 3)*: My first decision would be myself and then you know, the children and society. All that because like you're a father [speaking to another male participant], you've got a family.  *Male, focus group 2 (Pt ID 9)*: It [undergoing genomic test] might create awareness in them [family] also…..then you might encourage them to take those things, rather than waiting.  *Female, focus group 2 (Pt ID 13)*: I agree with [another participant]…..Once I started saying to them [children] about skin cancer…they have gone and got tested |
| Benefits beyond the individual | *Male, focus group 1 (Pt ID 6)*: Also something like this [genomics informed risk cancer screening] will basically benefit the whole society, right? Not just the few individuals, right. It's not about the, so it definitely will be great big impact because in long term it could affect plenty of people, to save lives basically  *Female, focus group 1 (Pt ID 3):* This is why all this research information is so good, because all the information that they'll get from us, it's all going to be put in studies. |
| Preferring not to know | *Male, focus group 2 (Pt ID 12)*: I’ve got a few friends that are sceptics and anti vaxxers and whatnot and that's their choice but like there is a smaller minority that are probably a bit sceptical about results and these sorts of tests and you know and aren't interested.  *Male, focus group 2 (Pt ID 9):* Some people say they prefer not to know certain things |
| Theme: Navigating the complexity of genomics | |
| Limited understanding of genomic testing | *Female, focus group 4 (Pt ID 22)*: Most people who get say a cancer diagnosis, they sort of go into shock and they're not really taking in the information that the doctor's giving them.  *Male, focus group 4 (Pt ID 25)*: Because most people don't understand risk.  *Male, focus group 3 (Pt ID 14):* I'm interested to know if anybody else has been overdiagnosed or overtreated… I'd be fascinated if they if they ever happened.  *Male, focus group 1 (Pt ID 6)*: I only mentioned that if it won’t be at the expense of the accuracy and the cost. Okay if it’s within obviously reasonable amount. Better is, I mean wider, better results  *Male, focus group 4 (Pt ID 25)*: I think we already know our genetics from our family histories and things like that.  *Female, focus group 4 (Pt ID 26)*: Because I can put my family history and see what the risks are… So why would I do a genomic test to see what my health risks are when I really already pretty good idea? |
| Information and result delivery needs to be clear and accessible | *Male, focus group 3 (Pt ID 14):* I like it online only because I have a brain injury and I sometimes I have to read things 3 or 4, 5 times to get the information through to my head and understand it.  *Male, focus group 1 (Pt ID 5)*: Yeah, I think it should be simple to understand. Like if it's too technical, too medical, it’s hard to understand for everyone.  *Male, focus group 2 (Pt ID 12)*: I just want to know my risk, how high it is, and how accurate it is for each of the different risks, of cancers or so.  *Male, focus group 3 (Pt ID 17)*: Yeah, I typically, you know support all the telehealth and you know virtual means of getting medical attention because I think it's most favourable for most people, especially the aged and people with disabilities.  *Female, focus group 4 (Pt ID 21)*: If something comes back [high risk result], I think you need to be guided by the health professional, so you don't get paranoid and think you're going to die and all this sort of stuff. |
| Theme: Trust in testing, providers and government | |
| The important of accuracy | *Male, focus group 3 (Pt ID 14):* I would have thought that the tests wouldn't have been made available unless they were extremely accurate, or at least to. Sort of like a like a 95% sort of thing or the in that area.  *Male, focus group 1 (Pt ID 6)*: It would be nice to get [a single test for multiple cancers], but we need to find some reasonable compromise, what you could realistically do here without losing accuracy? |
| Psychological impact of false positives | *Male, focus group 4 (Pt ID 23):* Accuracy is very important. I was screened for Alzheimer's, I was told I had it. It caused enormous distress in the family and I ultimately, I don't have it. I can't tell you the drama that it was involved in it.  *Female, focus group 2 (Pt ID 3):* Because I remember I had a test many, many years ago and it was actually a false test, not for this sort of thing, but for something else saying to do with, like, cervical cancer. And it was actually wrong, and I went through quite a bit of testing over two years…lot of screening and pretty invasive, wasn't very good. |
| Trust in healthcare providers through established relationships | *Female, focus group 4 (Pt ID 26):* I like to go to my GP because he knows us  *Female, focus group 3 (Pt ID 16)*: Not everybody has their own GP….and sometimes you don't have a strong relationship with a doctor you particularly trust, I'm afraid. |
| Concerns regarding healthcare provider skills and capacity | *Female, focus group 2 (Pt ID 8):* A re the GP's going to be educated about what this is [genomics informed risk cancer screening], why we’re doing it and what their role is?  *Male, focus group 2 (Pt ID 10):* I think if the nurse practitioner is actually trained in the genomics where they understand it, I would be okay with that. They'd have to have the training and a good understanding…  *Female, focus group 2 (Pt ID 13)*: I cannot see practise nurse sitting down going ohh, let's take a look at your genomic test. They're not trained to do that, in my opinion, I've had them in London and ohh god, they were useless really was basically just come in whip off a dressing  *Male, focus group 4 (Pt ID 23):* A new occupation has been created [genetic counsellors] (laughs) |
| Acceptability of screening recommendations | *Female, focus group 2 (Pt ID 8):* I would be [in response to being asked whether they would follow the recommendations for increased screening]. I’m a rule follower. Yes, so that’s just the way that I’m geared.  *Female, focus group 3 (Pt ID 15):* Even if I was told that I was low risk, I would still do the screening.  *Male, focus group 3 (Pt ID 14):* I’m exactly the same… I’d rather keep on top of the small percentage and have tests as regularly as I can, but it’s good to know that you’re at low risk.  *Female, focus group4 (Pt ID 21):* I’d still go on with my regular screening for the things that I do, I probably wouldn’t want to step back. I wouldn’t take it as gospel because things do crop up that you least expect….but I would hate to think I stepped back my screening and then that cost me my health |
